# MVGFormer: Multi-view perspective with graph-guided transformer for cryo-ET segmentation^★^

**DOI:** 10.1016/j.knosys.2025.114810

**Published:** 2025-11-12

**Authors:** Haoran Li, Xingjian Li, Huan Wang, Jiahua Shi, Huaming Chen, Yizhou Zhao, Bo Du, Johan Barthelemy, Daisuke Kihara, Jun Shen, Min Xu

**Affiliations:** a School of Computing and Information Technology, University of Wollongong, Australia; b ARC Training Centre for Innovative Composites for the Future of Sustainable Mining, University of Wollongong, Australia; c Centre for Nutrition and Food Sciences, Queensland Alliance for Agriculture and Food Innovation, The University of Queensland, Australia; d Ray and Stephanie Lane Computational Biology Department, Carnegie Mellon University, USA; e School of Electrical and Computer Engineering, University of Sydney, Australia; f Department of Management, Griffith University, Australia; g NVIDIA, USA; h Department of Biological Sciences, Purdue University, USA

**Keywords:** Cryo-electron tomography, Volumetric image segmentation, Deep learning

## Abstract

Cryo-Electron Tomography (cryo-ET) is a cutting-edge 3D imaging technology that enables detailed examination of biological macromolecular structures at near-atomic resolution. Recent deep learning applications on cryo-ET, such as cryo-ET segmentation, have drawn widespread interest for their potential to improve particle alignment, classification, and other tasks. However, current methods heavily rely on convolutional architectures, which prioritize local information while neglecting the global structural information inherent in cryo-ET data. Transformer-based models, known for their large receptive field, have become the de-facto design for 2D vision tasks due to their ability to effectively capture global information. This approach is also well-suited for 3D tasks, given the complex nature of 3D objects. Based on this, we extend 2D vision transformers into 3D and propose a novel transformer-based framework for cryo-ET segmentation, named MVGFormer. MVGFormer introduces a multi-view perspective fusion transformer encoder, which captures rich global structural information from multiple perspectives using unique positional embeddings. To enhance contextual awareness, we design a parallel context encoder that builds a visual graph to guide attention. We further introduce two complementary 3D decoders: multi-level feature fusion (MF) and parallel atrous convolutions (P3DA), which together capture multi-scale structural cues for precise segmentation. Furthermore, we introduce a view-masked self-supervised learning strategy to reinforce the effectiveness of the multi-view design and improve the model’s representation capability. To our knowledge, MVGFormer is the first transformer-based model for cryo-ET segmentation. We empirically evaluate MVGFormer on six cryo-ET datasets across three different tasks. Extensive experimental results demonstrate its superiority over state-of-the-art 3D segmentation methods.

## 1. Introduction

Cryo-Electron Tomography (cryo-ET) enables the rapid freezing and thinning of biological samples to an appropriate thickness, and facilitating imaging through an electron microscope [1]. Recent technical advances in cryo-ET allow it to capture high-resolution structures of macromolecular complexes in their near-native state [2], which is of great significance for studying the aspects of cell biology, biochemistry, and biomedicine. The analysis of cryo-ET plays an important role in facilitating the understanding of the virus infection mechanisms, drug discovery and disease treatment. However, due to the small size of each particle in the tomogram, it is difficult to fully observe them with the naked eye, leading to low efficiency in the field. Therefore, developing an automatic detection or segmentation model as an aiding approach or tool becomes crucial.

**Fig. 1.**
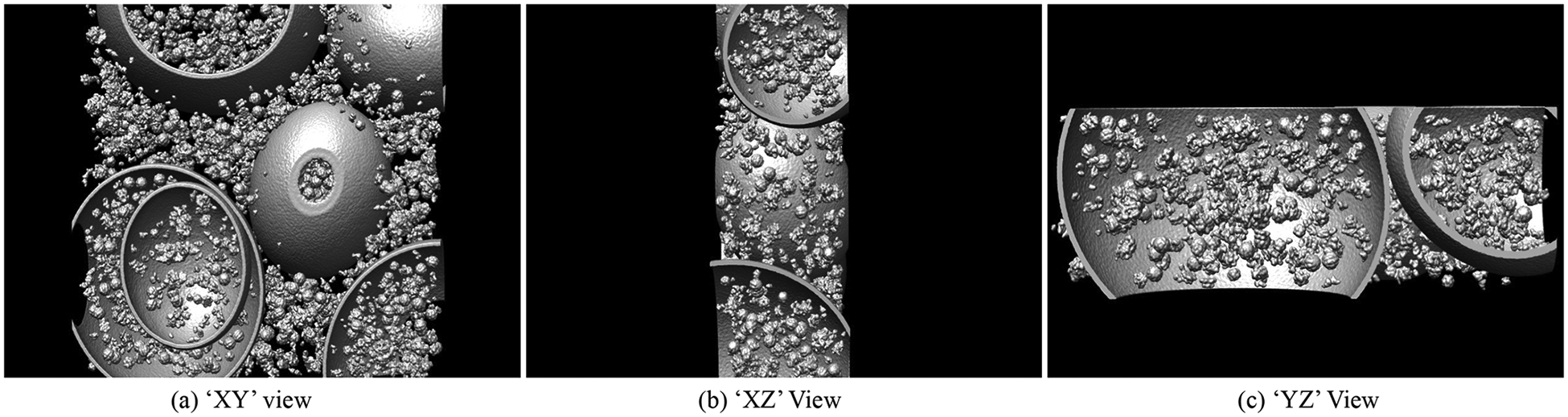
Example of the orthographic projection of a cryo-ET tomogram.

Different from 2D images, cryo-ET tomograms are featured by the volumetric data, which contains an extra dimension. Fig. 1 provides the orthographic projection of a cryo-ET tomogram. Cryo-ET tomogram is a grey-scale 3D image, typically represented in voxel space. Inspired by recent development of the deep neural network (DNN)-based approaches, some methods have been applied to processing cryo-ET images [3–6], especially in the field of cryo-ET segmentation. Cryo-ET segmentation can be divided into two sub-tasks: subtomogram segmentation and tomogram segmentation. Subtomogram segmentation aims to identify a target structure, typically a single macromolecule, within a subtomogram. Since most subtomograms in cryo-ET datasets contain one primary target of interest, the task is often formulated as a binary segmentation problem. In contrast, tomogram segmentation seeks to segment and classify all particles and other structures (e.g., membranes, filaments, compartments) within a whole tomogram, which usually contains thousands of particles and heterogeneous cellular components, making it similar to a multi-class semantic segmentation task. Although not originally designed for tomogram segmentation, several studies have applied existing deep learning models to cryo-ET tomograms and subtomograms [7], for example, for particle picking [8] or as part of simulation pipelines [9]. More recently, dedicated methods have been proposed for macromolecular segmentation [10] as well as for tomogram segmentation [11–13], the latter addressing a broader range of structures beyond macromolecules, such as membranes, filaments, and compartments. However, these methods are generally built upon 2D network architectures or conventional 3D U-Net backbones, which may limit their ability to fully capture the complex 3D spatial relationships in cryo-ET data.

Regrading the DNN-based approaches for 3D image segmentation, the existing models can be broadly categorized into two types based on their structural characteristics: convolutional neural network (CNN)-based and transformer-based. CNNs leverage their powerful inductive bias ability to gather local information at low-levels by using 3D convolutional kernels, while enabling the extraction of global information at high levels, and integrating information from different levels to obtain segmentation results through hierarchical structures [14,15]. However, compared to local information, global information is more crucial for three-dimensional data like tomograms, as their data distribution is more extensive. Different from CNN approaches, transformer-based approaches [16,17] capture global information through multi-head self-attention, which enlarge the receptive filed of the framework at the early stage. However, existing transformer-based architectures are primarily designed for 2D images. Even methods designed for 3D images, such as Magnetic Resonance Imaging (MRI), often involve dividing the input images into 2D slices for processing and deploy 3D reconstruction for post-processing. It tends to overlook the structural characteristics of the 3D data. Although a few other methods [18–20] have attempted to address this issue by focusing on voxel-level inputs, they all use the ‘XY’ view of the 3D image only as the input, unfortunately omitting the multi-perspective observation information of the 3D data. In fact, orthographic projections along the three principal axes provide complementary cues:the ‘XY’ view preserves the in-plane spatial organization, the ‘XZ’ view reveals vertical continuity along the depth axis, and the ‘YZ’ view captures lateral structures. Each individual view also suffers from limitations, e.g., the XY view lacks depth information, while the ‘XZ’ and ‘YZ’ views may distort in-plane spatial relations. By integrating these complementary perspectives, our multi-view design mitigates the ambiguities of single-view analysis and enables a more complete representation of cryo-ET data. Additionally, the recently developed Segment Anything Model (SAM) [21] for 2D images can serve as a foundation model for various tasks. Similarly, we aim to propose a 3D transformer architecture to provide a base framework for future cryo-ET foundation models.

With this insight, in this paper, we propose the **M**ulti-**V**iew Perspective with Graph-guided Transformer (**MVGFormer**), a multi-view perspective fusion transformer for cryo-ET segmentation. Our approach aims to fuse feature embedding from multi observation perspectives to fully leverage the characteristics of three-dimensional data. To further leverage contextual information to enhance segmentation performance, we employ a context encoder to generate a visual graph, which is then used to guide the attention process of the transformer. Additionally, inspired by recent 2D transformer-based segmentation approaches [22,23], we designed two types of 3D decoder to examine the feature presentations: multi-level feature fusion segmentor (MF) and parallel 3D atrous convolution segmentor (P3DA). MF obtains the final segmentation result by aggregating multi-level feature outputs from multiple stages of the transformer encoder. While P3DA restores the feature output from the last stage of transformer encoder to its original size and utilizes parallel 3D atrous convolutions with different dilation rates to gather multi-scale features. These features are then aggregated to generate the final segmentation mask. Furthermore, we conduct view-masked self-supervised learning to further validate the rationality and effectiveness of our multi-view design. To the best of our knowledge, this is the first work to extend vision transformers to cryo-ET segmentation task, and we believe the proposed MVGFormer can be served as a basic framework for the cryo-ET foundation models.

In a nutshell, our contributions are listed as follows:

- We propose MVGFormer, a multi-view perspective fusion framework with a dual-stream encoder that integrates contextual information through a visual graph to guide the transformer’s attention process.
- We design two different decoder variants to support MVGFormer: a multi-level feature fusion decoder (MF) that aggregates hierarchical features from multiple encoder stages, and a parallel 3D atrous convolution decoder (P3DA) that enhances multi-scale feature representation.
- To further enhance the effectiveness of the multi-view design, we introduce a view-masked self-supervised learning strategy, enabling the model to reconstruct masked views from remaining observations.
- We conduct extensive experiments to demonstrate the superior performance of MVGFormer over existing state-of-the-art cryo-ET segmentation methods.

## 2. Related work

### 2.1. Deep learning in cryo-ET

Deep learning approaches and their potential applications in cryo-electron tomography (cryo-ET) have been increasingly capturing the attention of the bioinformatics community. Some efforts have approached computer vision (CV) tasks and developed numerous new algorithms on cryo-ET data, i.e., segmentation [24–26], classification [27–29], and data augmentation [6,30]. Additionally, some researchers integrate cryo-ET with AI algorithms to address practical of processing cryo-ET data. Zeng et al. [2] proposes an unsupervised clustering approach for homogeneous structure mining and modeling. Liu et al. [31] introduces a dual-flow framework, which combines information from both tomograms and the generated mask for isotropic reconstruction. REST [32] introduces an U-Net based network to mine the relationship between the volumetric input and the ground-truth mask to enhance the model’s performance for the applications in cryo-ET (i.e.,article picking and subtomogram averaging). However, the aforementioned approaches are all convolutional structures-based, which have failed to effectively exploit the complex spatial information of the three-dimensional structure of cryo-ET. Hence, in this paper, we propose the first transformer-based framework (termed MVGFormer) for cryo-ET segmentation. Based on multi-view perspective transformer encoder and a multi-dilated rates atrous convolution decoder, our proposed MVGFormer can fully exploit the three-dimensional spatial information of cryo-ET.

### 2.2. Vision transformers

#### 2.2.1. 2D vision transformers

Transformers were first introduced to computer vision by Dosovitskiy et al. [16], who proposed the Vision Transformer (ViT) that transforms an image into a sequence of tokens and applies self-attention layers for classification. Due to its large receptive field and ability to capture global context at an early stage [33,34], ViT has achieved excellent results in many visual recognition tasks [35]. Inspired by ViT, many novel approaches have been proposed for downstream 2D vision tasks, i.e., classification [36–38], segmentation [22,23,39–41], and object detection [42–44]. To enhance data diversity, Wang et al. [45] replaces the fixed positional embeddings commonly used in ViT-based models with shuffled position embeddings, improving generalization across datasets.

#### 2.2.2. 3D vision transformers

With the success of transformers on 2D images, researchers have attempted to extend them to 3D volumetric data. Some studies [46–49] cut 3D images into multiple slices and treat them as inputs to deploy 2D transformers on 3D images. However, these approaches leave a significant portion of the spatial information in the third dimension uncomputed. To overcome this limitation, other methods [18,19] directly use voxel-level inputs to extend 2D transformers into 3D architectures. Although these methods have achieved promising results, they all assume a fixed observation perspective as the input view for the 3D image, neglecting the rich information observable from other perspectives. Motivated by this limitation, our work proposes a multi-view perspective fusion transformer encoder to fuse features from different observation perspectives to improve segmentation performance.

### 2.3. Multi-view fusion

Although a single-view image can provide sufficient information for model learning in 2D scenarios, in many complex situations, relying solely on the semantic information from one view is insufficient for the model to capture rich and comprehensive representations. PixelFusion-Net [50] fuses misaligned photographs of the same scene, significantly improving the network’s ability to learn large disparities. MVMP [51] achieves more efficient and faster person re-identification by integrating multi-view surveillance images. MFFN [52] fuses multi-view images obtained through flipping and enhancement of the input to more accurately identify the boundaries of camouflaged objects. EditSplat [53] leverages 3D Gaussian Splatting to fuse multi-view projections of objects in a 3D scene, enabling more view-consistent 3D scene editing. Unlike existing approaches, our method operates in the voxel space, processing different views of the same object to enable the model to learn richer three-dimensional contextual representations.

## 3. Problem definition

We tackle the cryo-ET segmentation task, which aims to predict the 3D mask for each particle in a voxel-level input image. The cryo-ET segmentation task can be divided into two sub-tasks: tomogram segmentation and subtomogram segmentation. For tomogram segmentation, the input is usually a large-scale 3D image (512^3^ in our unique experimentation data) in voxel-space and each input usually contains hundreds or thousands of particles. And the goal of tomogram segmentation is to segment and classify every particle contained in the input tomogram, similar to the semantic segmentation in 2D images. For subtomogram segmentation, the input is usually a small size 3D image (32^3^ in our case) in voxel-space and each input only contains very few particles and all the particles belong to the same category. The goal of subtomogram segmentation is to generate a 3D binary mask to localize each particle contained in the subtomogram.

Given an input cryo-ET tomogram/subtomogram $x\in R^{H\times W\times D\times 1}$, and the corresponding ground-truth segmentation mask $y\in R^{H\times W\times D\times N}$ (N denotes the number of the classes and N=1 for subtomogram input). The goal of cryo-ET segmentation task is to train a segmentation model which can obtain the most accurate predicted segmentation mask through

(1) $$\theta^{*}=\text{arg}\;\underset{\theta }{\text{min}}\;{ℒ}_{\text{CE}}\left(f_{\theta }(x),y\right),$$

where $\theta^{*}$ denotes the optimal model parameters, ${ℒ}_{\text{CE}}$ denotes the cross-entropy loss, y denotes the one-hot encoding of the label and $f_{\theta }(x)$ denotes the prediction probability from the model $f_{\theta }$.

## 4. Method

**Fig. 2.**
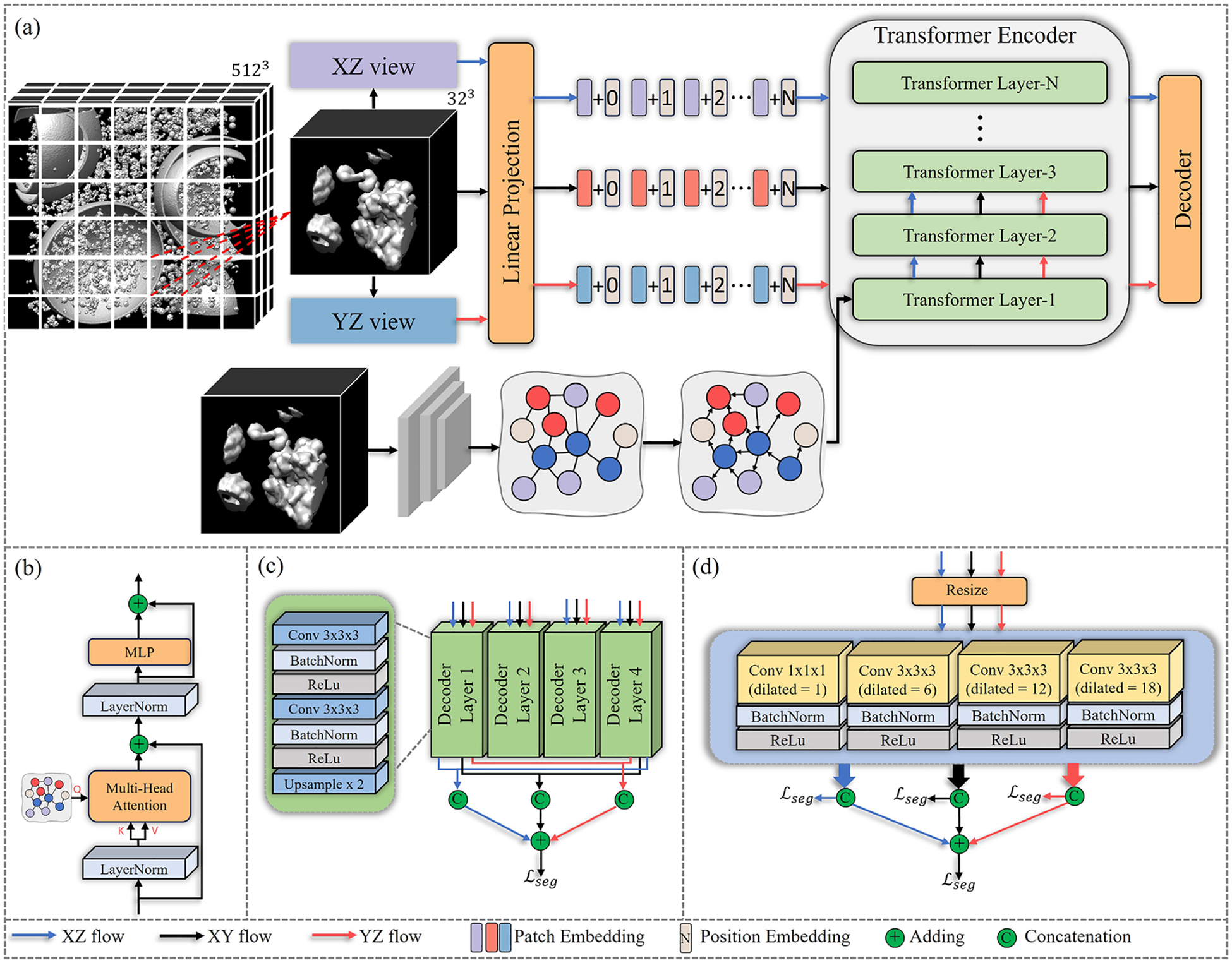
Overview of our proposed MVGFormer. (a) We cut the whole tomogram into patches and send each patch to the MVGFormer as the input. For each input, multi-view transform and linear projection are applied to obtain sequence-level feature embeddings from three different observation perspectives. Each feature embedding is added with its unique corresponding position embedding and then sent to transformer encoder. Each input is also sent to the context encoder to generate the visual graph as the attention-guidance. (b) is the structure of the transformer layer. To obtain voxel-level segmentation, we design two different decoders: (c) multi-level feature fusion segmentor and (d) parallel 3D atrous convolution segmentor. Best viewed in color.

As shown in Fig. 2, MVGFormer consists of a multi-view perspective fusion transformer encoder, a context encoder and a decoder to produce the segmentation mask. Given an input cryo-ET tomogram with size $H_{L}\times W_{L}\times D_{L}\times 1$, to reduce the computational load and increase the quantity of data, we first apply pre-processing to cut it into non-overlapping patches with size H×W×D×1 and send each patch as the input of the context encoder and the transformer encoder. Secondly, the decoder takes the output feature embedding from the encoders for final prediction. We propose two different decoder designs: multi-level feature fusion segmentor (MF) and parallel 3D atrous convolution segmentor (P3DA). Next, we present the details of the proposed encoder and decoders.

### 4.1. Context encoder

Given an input $x\in R^{H\times W\times D\times 1}$, we first send it to three convolutional blocks to obtain the context visual feature $f_{c}\in R^{C\times W_{p}\times H_{p}\times D_{p}}$. Each convolutional block comprises a 3×3×3 3D convolution layer, a batch normalization layer, and a ReLU activation. To facilitate the use of context features in guiding the transformer’s attention process, we construct a visual graph via k-means clustering on $f_{c}$, aiming to select more informative and representative graph nodes

(2) $$F_{c}=\left[f_{1},\ldots ,f_{j}\right]\in R^{C\times N},\;N=W_{p}H_{p}D_{p},$$

(3) $$\underset{\left\{\mu_{k}\right\},\text{R}}{\text{min}}\sum_{j=1}^{N}\sum_{k=1}^{K}R_{jk}{\left\|f_{j}-\mu_{k}\right\|}_{2}^{2},\;\text{s.t.}\;R_{jk}\in \{0,1\},\sum_{k=1}^{K}R_{jk}=1,$$

where $\mu_{k}\in R^{C}$ denotes the $k_{\text{th}}$ cluster center, K is a hyperparameter denotes the cluster number and $R_{jk}$ denotes the assignment of feature $f_{j}$ to cluster k

(4) $$R_{jk}=\left\{\begin{array}{ll} 1, & \text{if}\;k=\text{arg}\;{\text{min}}_{k^{\prime }}{\left\|f_{j}-\mu_{k^{\prime }}\right\|}_{2}^{2},\\ 0, & \text{otherwise.} \end{array}\right.$$

Hence, the graph node can be obtained through

(5) $$g_{k}=\frac{\sum_{j=1}^{N}R_{jk}f_{j}}{\sum_{j=1}^{N}R_{jk}},\;\text{for}\;k=1,\ldots ,K.$$

The graph nodes $𝒱={\left\{g_{k}\right\}}_{k=1}^{K}$ is further send to the transformer encoder for graph guided attention.

### 4.2. Multi-view perspective fusion transformer encoder

Existing 3D transformer works [18,19] only consider the ‘XY’ view of the input and set single position embedding from this input perspective, which overlooks the spatiality of the 3D images. As shown in Fig. 1(a–c), the information being observed varies from different perspectives. Hence, we propose the multi-view perspective transformer to fuse the features obtained from different observation angles. Different from traditional 2D transformers which treat images to be “isotropic” and use a single positional embedding, we regard the 3D images as “anisotropic”, which indicates the order of information acquisition differs depending on the perspective.

Given an input $x\in R^{H\times W\times D\times 1}$, we perform high-dimensional transpose to obtain the transposed inputs from ‘YZ’ view $x_{\text{YZ}}=x^{⊤}\in R^{H\times D\times W\times 1}$ and ‘XZ’ inputs from ‘YZ’ view $x_{\text{XZ}}=x^{⊤}\in R^{D\times W\times H\times 1}$. Then each input is divided into $L=\frac{H}{H_{p}}\times \frac{W}{W_{p}}\times \frac{D}{D_{p}}$ patches with each patch has a shape of $x_{p}\in R^{H_{p}\times W_{p}\times D_{p}\times 1}$. Those patches are further flattened and sent to a linear projection layer to obtain the sequence-level input $x_{\text{seq}}\in R^{C\times H_{p}W_{p}D_{p}}$, where C is the hidden dimension of the linear projection layer. As aforementioned, the information obtained from different observation perspectives will not share the same value, thus, we assign different learnable position embedding $p_{i}$ for $i_{\text{th}}$ position in the $x_{\text{seq}}$ obtained from different perspectives. Hence, the input $I_{\text{obv}}$ of the transformer can be formulated as:

(6) $$\begin{array}{r} I_{\text{obv}}=\left\{x_{1}^{\text{obv}}+p_{1}^{\text{obv}},x_{2}^{\text{obv}}+p_{2}^{\text{obv}},\ldots ,x_{C}^{\text{obv}}+p_{C}^{\text{obv}}\right\},\\ \text{obv}\in \left\{\text{XY},\text{XZ},\text{YZ}\right\}, \end{array}$$

where $x_{i}^{\text{obv}}$ denotes the input token embedding and $p_{i}^{\text{obv}}$ denotes the $i_{\text{th}}$ position embedding of the relevant observation perspective.

Different from existing 2D vision transformer [16,22], we set the graph nodes 𝒱 obtained from the context encoder as the Query to guide the attention process. Given an input embedding sequence $I_{\text{obv}}$, a $L_{n}$-layer transformer which contains Multi-head Self-Attention (MSA) and Multiplayer Perceptron (MLP) (Fig. 2(b)) is used to obtain the output feature embedding:

(7) $$F_{L_{1}}^{\prime }=\text{MSA}\left(Q=𝒱,K=\text{LN}\left(I_{\text{obv}}\right),V=\text{LN}\left(I_{\text{obv}}\right)\right)+I_{\text{obv}},\;F_{L_{n}}^{\prime }=\text{MSA}\left(\text{LN}\left(F_{L_{n-1}}\right)\right)+F_{L_{n-1}},\;n\geq 2,\;F_{L_{n}}=\text{MLP}\left(\text{LN}\left(F_{L_{n}}^{\prime }\right)\right)+F_{L_{n}}^{\prime },$$

where LN(⋅) represents the layer normalization. We denote $F_{\text{obv}}^{L_{n}}$ as the output sequence-level features of each layer from different observation perspectives.

### 4.3. Decoder designs

As mentioned in Section 1, two different decoder designs are introduced for voxel-level segmentation. We set the cross-entropy loss ${ℒ}_{CE}$ as the segmentation loss ${ℒ}_{\text{seg}}$ [54–56] for both decoder designs.

#### 4.3.1. Multi-level feature fusion segmentor (MF)

As shown in Fig. 2(c), we propose a multi-level feature fusion segmentor to aggregate N feature embeddings obtained from different encoder layers. Given a $L_{n}$-layer transformer encoder, MF takes the output features from the $\left\{{\frac{L}{N}}_{th},2{\frac{L}{N}}_{th},\ldots ,N{\frac{L}{N}}_{th}\right\}$ layer as the input, and reshapes each feature to $F_{N}\in R^{C\times H_{p}\times W_{p}\times D_{p}}$. Each $F_{N}$ is further sent to a separate decoder blocks, which consists of two 3×3×3 convolution layers, two Batch Normalization (BN) layers, two rectified linear unit (ReLU)-layers and one up-sampling layer. Then we concatenate all N outputs from the decoder blocks and use a 1×1×1 convolution layer to generate the segmentation mask from different observation perspectives ${\overset{ˆ}{y}}_{\text{obv}}$. Consequently, we add those predicted masks together to obtain the final prediction mask

(8) $$\overset{ˆ}{y}={\overset{ˆ}{y}}_{\text{XY}}+{\overset{ˆ}{y}}_{\text{XZ}}^{⊤}+{\overset{ˆ}{y}}_{\text{YZ}}^{⊤}.$$

It should be noted that the predicted masks from the ‘YZ’ view, ${\overset{ˆ}{y}}_{\text{YZ}}\in R^{H\times D\times W}$, and the ‘XZ’ view, ${\overset{ˆ}{y}}_{\text{XZ}}\in R^{D\times W\times H}$, are rearranged into the canonical (H×W×D) grid before the adding operation, so that they are spatially aligned with ${\overset{ˆ}{y}}_{\text{XY}}\in R^{H\times W\times D}$. Since all views share the same spatial resolution and are normalized in the joint latent space, their features are comparable in scale and semantics. We therefore adopt element-wise addition for fusion, which reinforces complementary cues across views while remaining more lightweight and effective than concatenation + MLP, cross-attention, or FiLM-style gating.

#### 4.3.2. Parallel 3D atrous convolution segmentor (P3DA)

We present our proposed P3DA in Fig. 2(d). P3DA takes the output feature $F_{L_{n}}$ from the final layer of the transformer encoder and reshape it to the original size H×W×D as the input 3D feature map $F\in R^{C\times H\times W\times D}$. F is further sent to four parallel atrous decoder blocks. Each block consists of a 3×3×3 convolution layer (except the first block uses a 1×1×1 Conv) with different dilated rates to enlarge the receptive field [57,58], a BN layer and a ReLU layer. The dilation rates for four blocks are set to 1, 6, 12 and 18. Then, through a concatenation operation, the parallel output of the decoder layers can be fused and sent to a 2-layer (both with kernel size 1×1×1) to produce the segmentation mask. Similar to MF, we perform the same adding operation on the predicted masks ${\overset{ˆ}{y}}_{\text{obv}}$ from different observation perspectives to obtain the final result $\overset{ˆ}{y}$.

### 4.4. View-masked self-supervised learning (VSL)

To facilitate a deeper understanding of inter-view relationships, we introduce a novel self-supervised learning strategy aimed at improving segmentation performance. In details, each time during training, we randomly select one input view as the masked view, and reconstruct the masked view from the remaining two views. Taken $I_{\text{XY}}$ as an example, we randomly mask $I_{\text{XY}}$ with a rate η and then train the remaining part with the rest two views following the standard procedure described above. Then, following [59], we introduce an additional lightweight transformer as the decoder to reconstruct the masked regions, and use the mean squared error (MSE) loss ${ℒ}_{\text{MSE}}$ as the reconstruction loss ${ℒ}_{\text{recon}}$.

### 4.5. Optimization

We separately calculated the segmentation loss for the predicted mask ${\overset{ˆ}{y}}_{\text{obv}}$ from each observation perspective, as well as for the fused segmentation mask $\overset{ˆ}{y}$. Hence, the target function for training can be formulated as

(9) $$ℒ={ℒ}_{\text{seg}}\left(y,{\overset{ˆ}{y}}_{\text{XY}}\right)+{ℒ}_{\text{seg}}\left(y,{\overset{ˆ}{y}}_{\text{YZ}}^{⊤}\right)+{ℒ}_{\text{seg}}\left(y,{\overset{ˆ}{y}}_{\text{XZ}}^{⊤}\right)+{ℒ}_{\text{seg}}(y,\overset{ˆ}{y})+{ℒ}_{\text{recon}},$$

where y denotes the ground-truth mask. To help better understanding the proposed framework, we also include a concise overview of each component in Table 1.

## 5. Experiments

### 5.1. Experimental settings

#### 5.1.1. Datasets

We employ two types of datasets: cryo-ET tomogram dataset and cryo-ET subtomogram dataset.

##### Tomogram dataset.

We chose the tomogram dataset used in SHREC2021 [60] as the tomogram dataset. This dataset contains 10 cryo-ET tomograms simulated from 13 proteins (see details in Appendix A) of known structure with varying sizes, shapes and functions. To plus vesicle and fiducial, each tomogram contains 15 types of particles, and has a shape of 512^3^. Following [60], we set the first nine tomograms as the training set and the last tomogram as the test set. Due to the size of each input tomogram is too large for training, we cut each tomogram into multiple non-overlapping patches with size 32^3^, and use each patch as the input for the model. Hence, there are total 40,960 samples in the SHREC dataset (36,864 samples in training set and 4096 samples in test set).

To further validate the generalization ability of our method on real tomograms, due to the scarcity of datasets, there is currently no publicly available real Cryo-ET tomogram dataset with voxel-level annotations. Hence, we employed the particle picking task, an instance of voxel-level segmentation, to quantitatively evaluate the performance of MVGFormer on real Cryo-ET tomograms from the EMPIAR-10499 dataset [61] and CZII [62]. EMPIAR-10499 contains 65-tilt series of native M.pneumoniae cells with annotated ribosomes. CZII is an open challenge dataset which contains 6 particle types with different difficulty levels of prediction.

##### Subtomogram dataset.

**Fig. 3.**
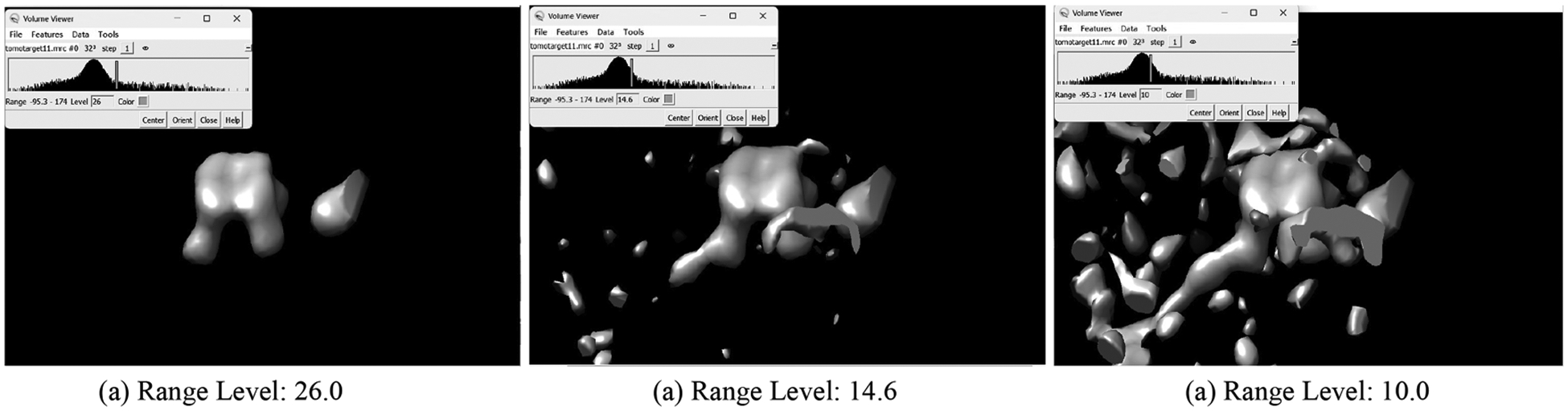
Example visualizations of the input cryo-ET subtomogram using UCSF Chimera with different filtering ranges.

Both simulated dataset and real dataset are used for experimental studies. Simulated dataset is generated following the same process as [24,64], which explicitly incorporates the tomographic reconstruction procedure with missing wedge effects and the contrast transfer function (CTF) [65,66]. For illustration, an example visualization of the input simulated data is provided in Fig. 3. The whole dataset contains 50 macromolecules (see details in Appendix A) and each macromolecule is simulated with three different noise levels, with SNR at 0.03, 0.05 and Infinity. Each noise level contains 500 samples. The simulated dataset contains 75,000 subtomograms in total. For real dataset, public dataset Poly-GA [67] and Erwinia [68] are chosen. PolyGA contains 1033 samples in total (66 26*S* subtomograms, 66 *T RiC* subtomograms and 901 *Ribosome* subtomograms). Following [69,70], all the input subtomograms are resized to 32^3^, and the dataset is randomly split into training set and test set with the ratio of (0.85 : 0.15). The ground-truth segmentation mask of Poly-GA is provided by [24,69], and we extracted the segmentation ground-truth masks for each subtomogram based on the coordinate locations provided in Guo et al. [67]. Erwinia contains 10 samples in total. The size of each subtomogram ranges from 72^3^ to 84^3^. Following [71], we resize each subtomogram to 64^3^ and partitioned it into multiple non-overlapping patches of size 32^3^. Each patch is then used as an input to the model, resulting in a total of 80 samples from the Erwinia dataset.

#### 5.1.2. Implementation details

We train our model on two NVIDIA A100 Tensor Core GPUs with a 80GB memory per card. For training, the layer number $L_{n}$, hidden dimension C, attention head number and mask rate η are set to 12, 256, 16 and 50%. The cluster number K is set to 16. The patch size $\times H_{p}\times W_{p}\times D_{p}$ is set to 4×4×4. We choose the Adam optimizer [72] with the initial learning rate set to 1e–3. The model is trained for 200 epochs with batch size 72 and the learning rate is decayed 90% for every 100 epochs.

#### 5.1.3. Evaluation metrics

Following existing volumetric segmentation approaches, we choose the mean intersection of union (mIoU) and dice similarity coefficient (Dice) as the core evaluation metrics. For particle picking task, precision, recall, and F_1_ score are set as the evaluation metrics.

#### 5.1.4. Baselines

For tomogram segmentation, following [60], we set URFinder [60], U-CLSTM [73], MCDSNet [60], YOPO [60], CFN [60], TM-F [74], TM [74] and DeepFinder [8] as baselines. Besides, we add a commonly used voxel-level segmentation method VoxResNet [75] and three recent methods, MedNeXt [18], Swin UNETR [20] and SwiFT [19], as additional baseline methods. VoxResNet is a 3D convolution-based model designed for volumetric image segmentation. MedNeXt is hierarchical transformer architecture for CT and MRI modalities. Swin UNETR and SwiFT both extend 2D Swin transformer into higher dimension for segmentation. For tomogram particle picking, we set DeepFinder, crYOLO [76], EMAN2 [77], VoxResNet, and SwiFT as the baselines. For subtomogram segmentation on simulated dataset, we use U-CLSTM, DeepFinder, VoxResNet, Swin UNETR, MedNeXt and SwiFT as baselines. For subtomogram segmentation on real dataset, due to the small scale of the real dataset, we utilize VoxResNet pre-trained on simulated subtomogram dataset as the baseline.

### 5.2. Comparisons with state-of-the-art methods

#### 5.2.1. Tomogram segmentation

The comparisons on SHREC2021 dataset are shown in Table 2. We report the results using both MVGFormer (MF) and MVGFormer (P3DA). For a fair comparison, we use the same training set for the training of VoxResNet, MedNeXt, Swin UNETR and SwiFT. All the results reported are the average results of five training runs, avoiding the occurrence of the randomness in results. We also include the standard deviation in the table. The results of other baselines are directly cited from their papers. As can be seen, our MVGFormer (MF) and MVGFormer (P3DA) perform better than all baselines. Compared with VoxResNet, our methods excels on both *mIoU* (i.e., 83.7% → 85.7% for MVGFormer (MF) and 83.7% → 86.9% for MVGFormer (P3DA)) and *Dice* (i.e., 91.1% → 92.6% for MVGFormer (MF) and 91.1% → 93.1% for MVGFormer (P3DA)). This is because our transformer encoder enables our model to gather global information at early stage to acquire more abundant structural information of the 3D data. Compared with SOTA transformer-based 3D segmentation approaches, MedNeXt and SwiFT, our methods still outperforms on all metrics (i.e., *mIoU* increased by 5.1% and *Dice* increased by 3.7%). This is due to the fact that they only consider the input from the ‘XY’ perspective, overlooking the spatiality of the information contained in 3D images. And we also compare the two decoder designs we proposed in Table 2. Because of the atrous convolutions can enlarge the receptive field and better capture contextual information, MVGFormer (P3DA) achieves better performance compared to MVGFormer (MF) (i.e., 84.7% → 86.9% in *mIoU* and 81.7% → 93.1% in *Dice*). Although the MF variant itself already outperforms existing baselines, it is mainly included as an ablation design, while our final model consistently adopts P3DA as the segmentation decoder due to its stronger and more robust performance. To break the strict voxel-wise correspondence among views, We have added a permutation by introducing Gaussian noise (σ=0.05) to the XZ-view inputs and perform the same experiment on the SHREC2021 dataset. As can be seen from Table 2, the permutation-based baseline’s performance remained comparable to the unperturbed setting, indicating that the model captures high-level semantic relationships rather than simple spatial re-indexing.

**Fig. 4.**
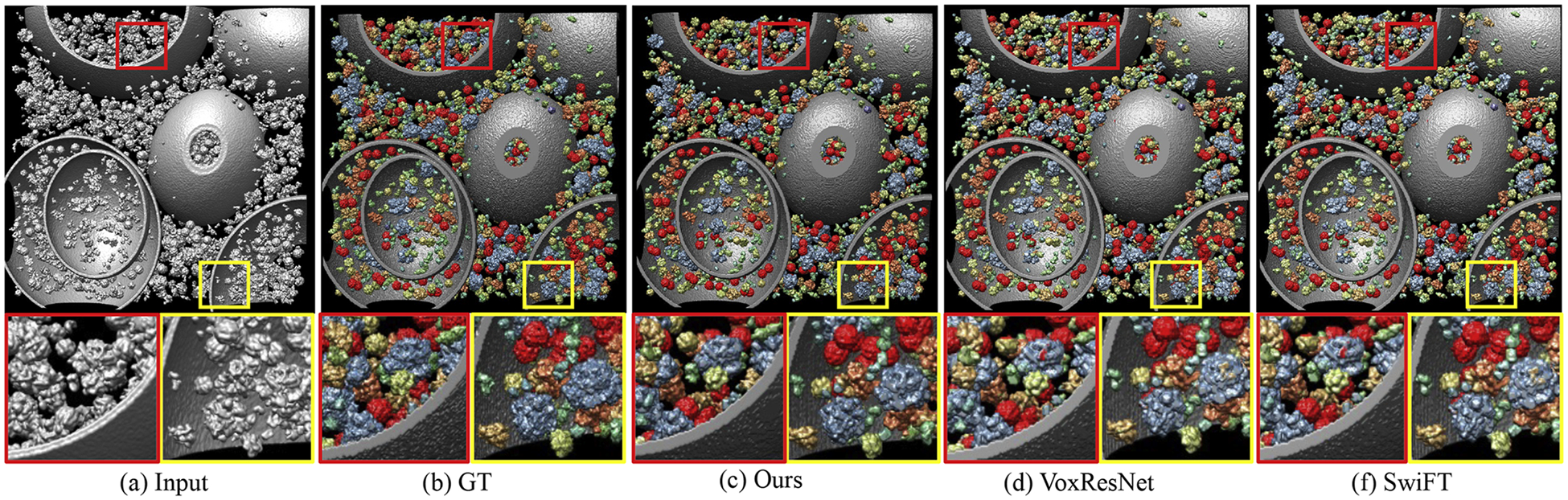
Visualization of cryo-ET segmentation results. The boxes in the first row indicate the areas that the close-ups on the bottom display. The 3D cryo-ET visualization results are obtained through UCSF Chimera [63].

**Fig. 5.**
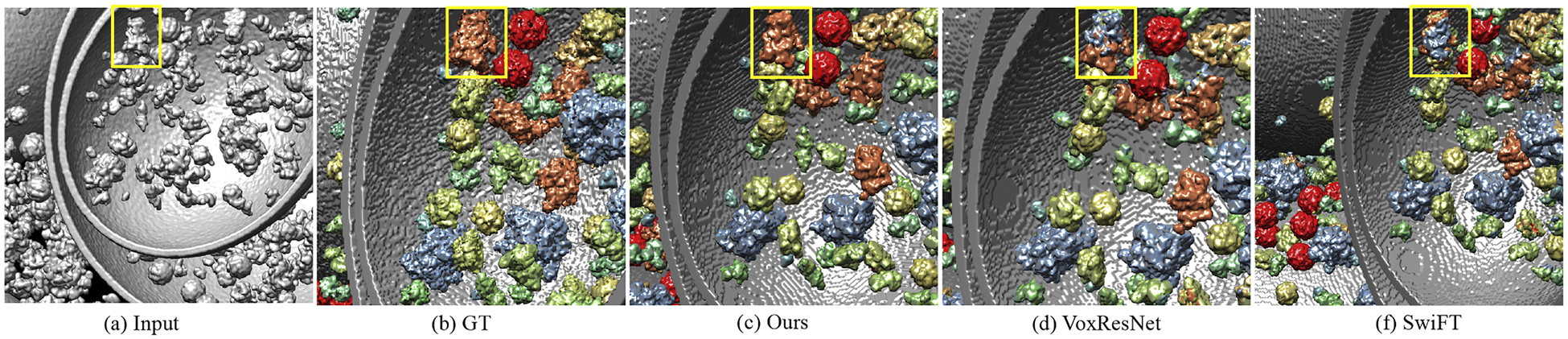
Magnified close-ups of the cryo-ET segmentation results. A ‘1QVR’ protein particle is located within the yellow box.

Fig. 4 shows the segmentation results of tomogram on the SHREC2021 dataset. To mitigate potential inconsistencies at patch borders, we employ an overlap-tile inference strategy rather than non-overlapping tiling. Specifically, during inference the tomogram is divided into 32^3^ subvolumes with a 50% overlap in each dimension. The network outputs per-voxel class probabilities for each subvolume, and predictions in overlapping regions are fused by weighted averaging using a smooth window function. For better visibility, we enlarged two local parts as close-ups and outlined them with yellow and red boxes respectively in the original figures. We provide the segmentation results from MVGFormer (P3DA) as ours. Compared to baselines, our proposed method demonstrates superior performance in terms of segmentation details. In contrast, it can be seen from the yellow boxes that both VoxResNet and SwiFT incorrectly segment some proteins of type “5MRC” as type “1BXN” (the color of the specific part on the mask should be blue instead of yellow). It is obviously shown on the red boxes that both VoxResNet and SwiFT inaccurately classify some proteins of type “5MRC” as type “4V94” (in the center part, where color blue is wrongly marked by red). To better showcase the segmentation results, we also provide close-up magnified visualizations in Fig. 5.

Inherently the classical transformer architecture lacks the strong inductive bias of convolutional structures, and thus requires a large amount of training data to achieve superior performance (i.e., 2D transformers typically use ImageNet for training, which contains approximately 1,300k training samples.). However, in cryo-ET, such large amounts of annotated image data are scarce. The 40k sample cryo-ET tomogram dataset SHREC and the 75k simulated subtomogram dataset used in our research are the largest known tomogram and subtomogram dataset with reliable annotations. Given that subtomograms and local regions of tomograms share the same imaging characteristics (voxel size, CTF, noise distribution, and missing-wedge geometry) and exhibit highly similar structural patterns for the target particles. Therefore, pre-training on subtomograms enables the model to learn particle-level structural and feature representations that transfer effectively to particle segmentation within whole tomograms. Therefore, to further prove the effectiveness of our proposed MVGFormer our larger scale datasets, we conduct an additional experiment by pre-training the proposed network using the simulated subtomogram dataset, and then fine-tuned the pre-trained encoder on the tomogram dataset. In detail, the model is pre-trained for 100 epochs on the simulated dataset with a batch size of 72 and a learning rate of 1e-3. After pre-training, we fully fine-tuned the best-performing checkpoint on the SHREC2021 dataset. The results are shown in Table 4. As can be seen from the table, pre-training on the large-scale simulated subtomogram dataset effectively increases the diversity and amount of data seen by the model, leading to improved performance of our proposed method. Compared to the baseline methods, the proposed MVGFormer shows a substantial improvement, with mIoU increasing by 5.5% and Dice rising from 91.4% to 95.1%.

#### 5.2.2. Tomogram particle picking

Due to the absence of voxel-level annotations, we follow the standard practice in Cryo-ET tomogram analysis [78–80], where the predicted particle center positions are compared with the known center positions. A particle is considered correctly detected (true positive) if the Euclidean distance between the predicted and known centers is less than the particle radius. We train all the baselines and the proposed MVGFormer on the PolyGA dataset, as it contains voxel-level mask annotations of ribosome particles. Subsequently, we test all the methods on the EMPIAR-10499 dataset and CZII dataset, and report the average results in Table 5. As shown in the table, our proposed method outperforms a wide range of state-of-the-art models. For instance, MVGFormer (P3DA) achieves 61.2%, 78.4% and 69.8% in terms of Precision, Recall and F_1_ score, surpassing the VoxResNet by 7.2%, 9.8% and 7.9% on the EMPIAR-10499 dataset. And on CZII dataset, our proposed MVGFormer (P3DA) still demonstrates outstanding performance (i.e., Precision increased by 15.8% compared to SwiFT and $F_{1}$ increased by 8.7% compared to VoxResNet). The results demonstrate that the proposed MVGFormer exhibits excellent generalization and adaptability on real Cryo-ET tomograms.

To further explore the generalization ability of our model on zero-shot tasks, we directly apply the model pretrained on the SHREC dataset to the particle picking task on EMPIAR-10499 and CZII datasets, and reported the results in Table 6. While the P3DA decoder offers larger receptive fields and richer contextual representations, its higher parameterization makes it prone to overfitting the source domain. In contrast, the lightweight MF design provides stable feature refinement with fewer parameters, which is particularly beneficial under zero-shot conditions where domain gaps are prominent. As shown in the table, our method (MVGFormer(MF)) achieves 53.2%, 63.5% and 57.9% in terms of Precision, Recall and F_1_ score on the EMPIAR-10499 dataset, and 69.6% Precision, 63.7% Recall, and 66.5% F_1_ on the CZII dataset, outperforming baseline methods and demonstrating promising generalization capability to unseen tasks.

#### 5.2.3. Subtomogram segmentation

**Fig. 6.**
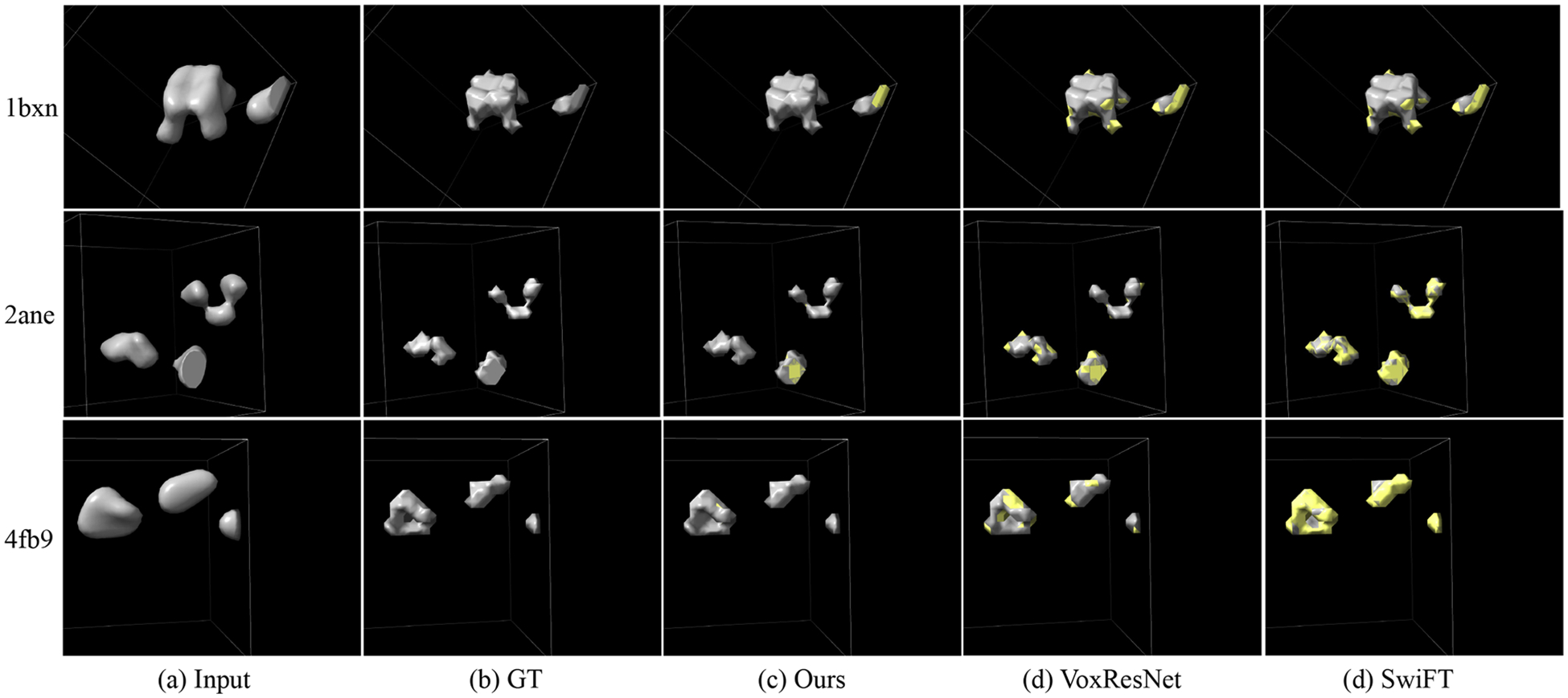
Visualization of cryo-ET subtomogram segmentation results at an SNR of 0.03. Regions of disagreement between the prediction and the ground truth are highlighted in yellow as error annotations. Best viewed in color.

**Fig. 7.**
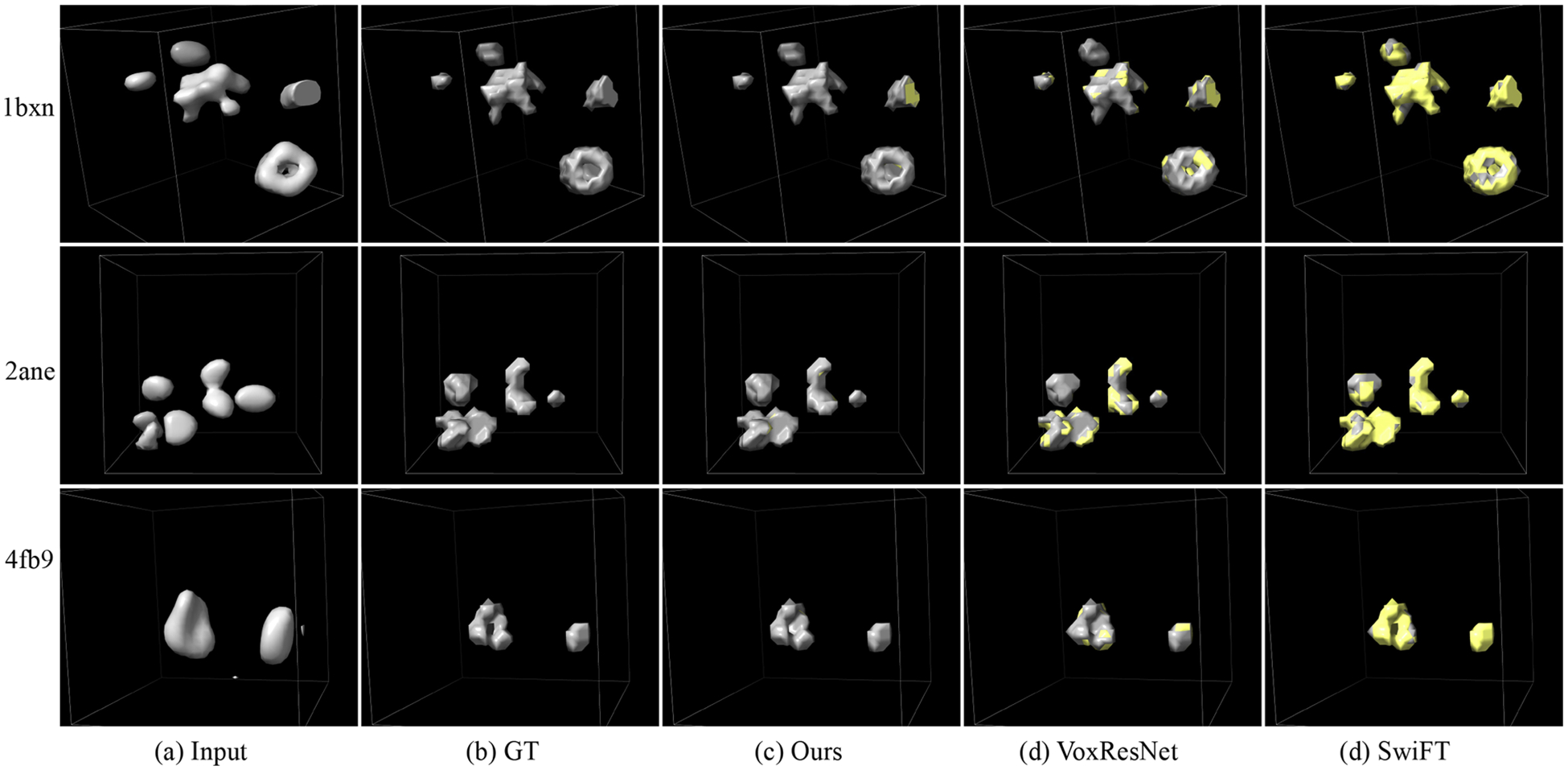
Visualization of cryo-ET subtomogram segmentation results at an SNR of 0.05. Regions of disagreement between the prediction and the ground truth are highlighted in yellow as error annotations. Best viewed in color.

**Fig. 8.**
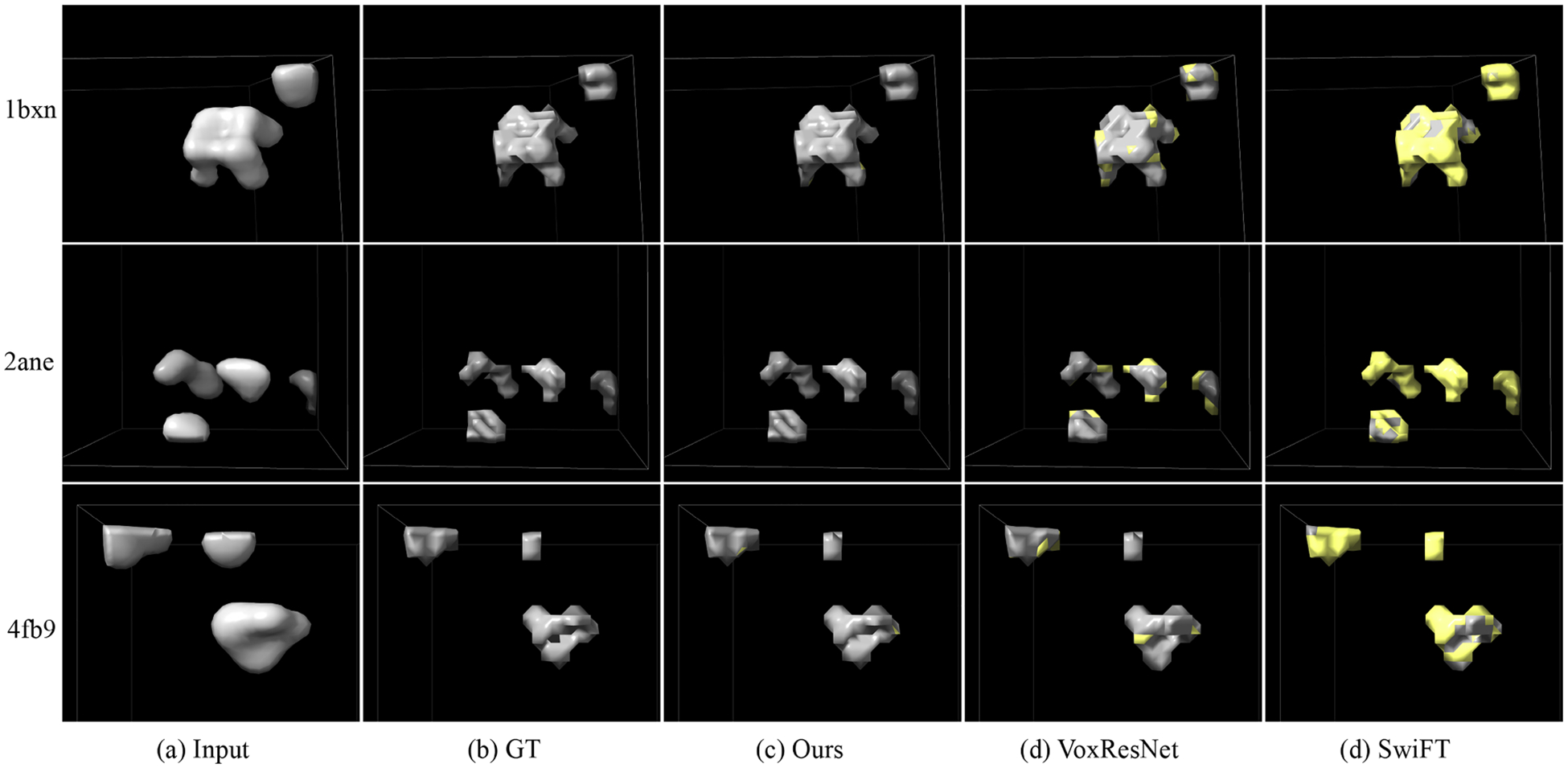
Visualization of cryo-ET subtomogram segmentation results with the noise of Infinity SNR. Regions of disagreement between the prediction and the ground truth are highlighted in yellow as error annotations. Best viewed in color.

Table 3 presents the segmentation results on both the simulated and real subtomogram datasets. Same to the tomogram segmentation, we also report the average results of five training runs. As can be seen, both MVGFormer (MF) and MVGFormer (P3DA) achieve SOTA performance compared to other baselines. For the results on simulated dataset, our MVGFormer (P3DA) leads to substantial performance gains in *mIoU* (i.e., 80.1% → 87.1% compared to SwiFT) and *Dice* (i.e., 91.4% → 93.2% compared to VoxResNet). We also provide the visualization of subtomogram segmentation results with the noise of 0.03 SNR, 0.05 SNR and Infinity SNR in Figs. 6, 7 and 8. As can be seen, our MVGFormer performs better in segmenting certain details (i.e., at the upper right corner particle of ‘2ane’, our segmentation result clearly adhere more closely to the ground truth mask; and for ‘4fb9’, compared to VoxResNet, MVGFormer fully segments out the particle on the left side, while VoxResNet leaves a gap in the middle part of the particle.).

For the results on the real datasets, due to the transformers lack the strong inductive bias of convolutional networks, they cannot directly exhibit good performance on small datasets [16]. As mentioned in Section 5.1.2, we use the MVGFormer (P3DA) pre-trained on the simulated dataset as the basic network and fine-tune it on the real dataset to test the performance of our model on the test set. We provide both pretrain VoxResNet and VoxResNet without pre-training (VoxResNet (w/o pre)) as the baselines. As shown in Table 3, our method yields 63.7% in *mIoU* and 78.5% in *Dice*, surpassing the other two baselines on both metrics. The experimental results in Table 3 on Erwinia dataset demonstrate the strong and consistent performance of our method (i.e., *mIoU* increased by 6.7% compared to SwiFT and *Dice* increased by 1.3% compared to VoxResNet), highlighting its robustness across different data domains.

#### 5.2.4. Computational cost

We report the computational cost of the proposed MVGFormer and the three most comparable baselines in Table 7. Compared to purely convolutional methods (VoxResNet), the proposed transformer-based approach has more parameters indeed and requires somewhat longer training time. However, almost all transformer methods involve a trade-off between training cost and performance, whereas achieving performance improvements at the expense of higher training costs. Besides, during the inference stage, our method achieves a significant performance improvement with only a slight increase in inference time compared to MedNeXt and SwiFT.

### 5.3. Ablation studies

#### 5.3.1. Hyper-parameter selection

We further evaluate the hyper-parameters in our approach. As shown in Table 8, we evaluate the size of the hidden dimension C, the number of the transformer layer $L_{n}$ and the patch size $H_{p}\times W_{p}\times D_{p}$. As can be seen, the performance decreases when the hidden dimension C increases (e.g., *mIoU* decreased 3.5% compared to C=512 and *Dice* decreased 3.0% compared to C=128). Although the model achieves decent performance when C=1024, it comes with an increase in computational complexity. Similarly, the other two parameters, $L_{n}$ and $H_{p}\times W_{p}\times D_{p}$, will also simultaneously affect both the performance and the computational cost of the model. And the higher or lower values of these two parameters will not improve the model’s performance (i.e., 92.0% → 86.9% in *mIoU* compared to $l_{n}=6$ and 85.3% → 54.8% in *Dice* compared to $H_{p}\times W_{p}\times D_{p}=16\times 16\times 16$). Hence, the most competitive performance is provided by setting $C=256,L_{n}=12$ and $H_{p}\times W_{p}\times D_{p}=4\times 4\times 4$. We further evaluate the effect of the number of cluster centers K, as reported in Table 9. To verify the generality of this choice, additional ablation studies are conducted on four datasets covering tomogram segmentation, subtomogram segmentation, and particle picking tasks, with the results summarized in Table 10. The experimental results show that deviations in the number of cluster centers negatively impact the model’s performance. Decreasing or increasing any of these three parameters will lower the model’s performance, especially when increasing them, which also increases the computational complexity.

#### 5.3.2. Effectiveness of the multi-view perspective fusion strategy

We analyze the effectiveness of the proposed multi-view perspective fusion strategy (MVP). As aforementioned, existing transformer-based methods underperforms due to they only consider single observation perspective of the 3D image as the input. And the superiority of our approach partly comes from considering inputs from different perspectives and aggregate the output features. Hence, we deploy the MVP strategy to three recent 3D transformer-based approaches Swin UNETR, MedNeXt and SwiFT and re-conduct the experiments on the SHREC dataset. We report the results of both with and w/o MVP in Table 11. After incorporating our proposed MVP strategy, all three methods’ performance improved on both evaluation metrics (i.e., 81.5% → 83.4% in *mIoU* for SwiFT, and 90.5% → 91.2% in *Dice* for MedNeXt). This indicates that our designed MVP strategy indeed enhances model performance by aggregating multi-perspective information.

Additionally, to demonstrate that cryo-ET data is indeed anisotropic, we train each perspective as an independent primary viewpoint and provide the results in Table 12. Since each embedding is optimized separately, they learn different spatial priors that reflect the inherent anisotropy and spatial context of each view. As shown in the table, the proposed MVP strategy outperforms the use of individual viewpoints (i.e., 82.7% → 85.3% in *mIoU*), indicating that incorporating different views with distinct positional embeddings effectively captures diverse spatial priors. Hence, the results prove that different from 2D images, 3D images exhibits anisotropic characteristics, as mentioned in Section 4.2.

We further provide the ablation study using repeated single-view inputs with distinct positional embeddings in Table 13, and the feature diversity across different views in Table 14 using cosine similarity (CosSim) and the normalized feature diversity scores (FDS) as evaluation metrics.

(10) $$\text{CosSim}(\text{v}1,\text{v}2)=\frac{{\text{f}}^{\left(\text{v}1\right)}\cdot {\text{f}}^{\left(\text{v}2\right)}}{\left\|{\text{f}}^{\left(\text{v}1\right)}\right\|\left\|{\text{f}}^{\left(\text{v}2\right)}\right\|},$$

(11) $$\text{FDS}(\text{v}1,\text{v}2)=\frac{1}{N}\overset{N}{\underset{i=1}{\sum }}{\left\|{\text{f}}_{i}^{\left(\text{v}1\right)}-{\text{f}}_{i}^{\left(\text{v}2\right)}\right\|}_{2}^{2}.$$

As can be seen from Table 13, simply increasing the input dimensionality by duplicating a single view does not lead to significant performance improvements. In contrast, our multi-view perspective fusion (P3DA) achieves clear gains in both mIoU and Dice, confirming that the improvements stem from the integration of complementary information across views rather than from trivial input expansion. And the results from Table 14 show lower cosine similarity and higher diversity scores across different view-pairs, indicating that the views provide complementary structural cues. Together, these findings validate the effectiveness of our multi-view design in leveraging structurally diverse perspectives for improved representation learning.

#### 5.3.3. Effectiveness of the design choices

**Fig. 9.**
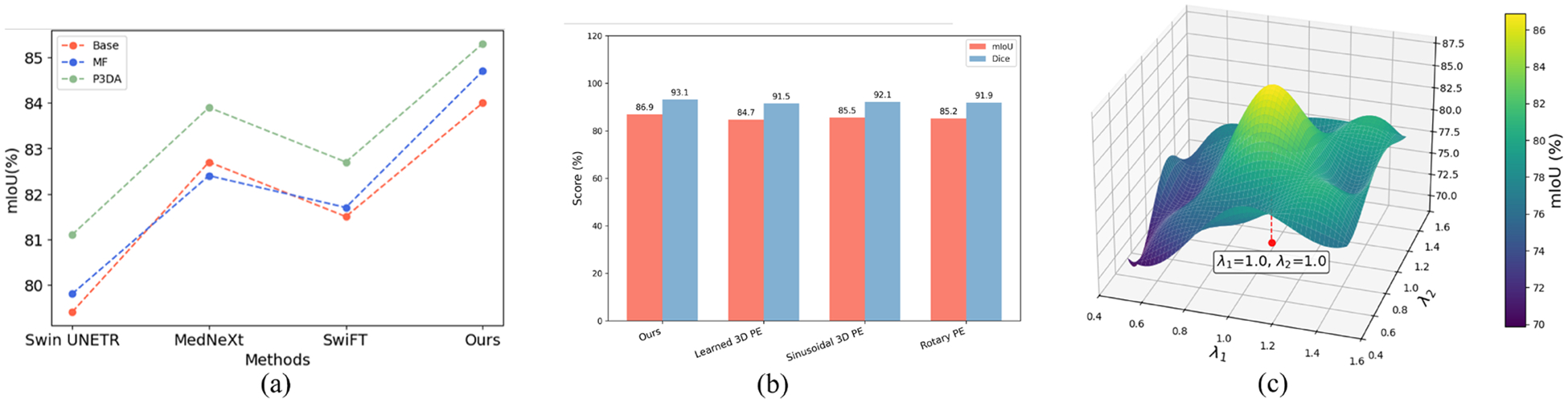
Ablation studies of (a) The proposed decoder designs. We replace the decoders of three recent 3D transformer-based approaches with our proposed decoders. (b) Different positional encoding design. (c) Loss balancing. We set different weight $\lambda_{1}$ and $\lambda_{2}$ for different losses.

We conduct comprehensive ablation experiments to assess the influence of different design choices, including graph construction methods, fusion schemes, loss function design and the decoder design, and report the results in Table 9 and Fig. 9.

##### Effectiveness of the graph design.

**Fig. 10.**
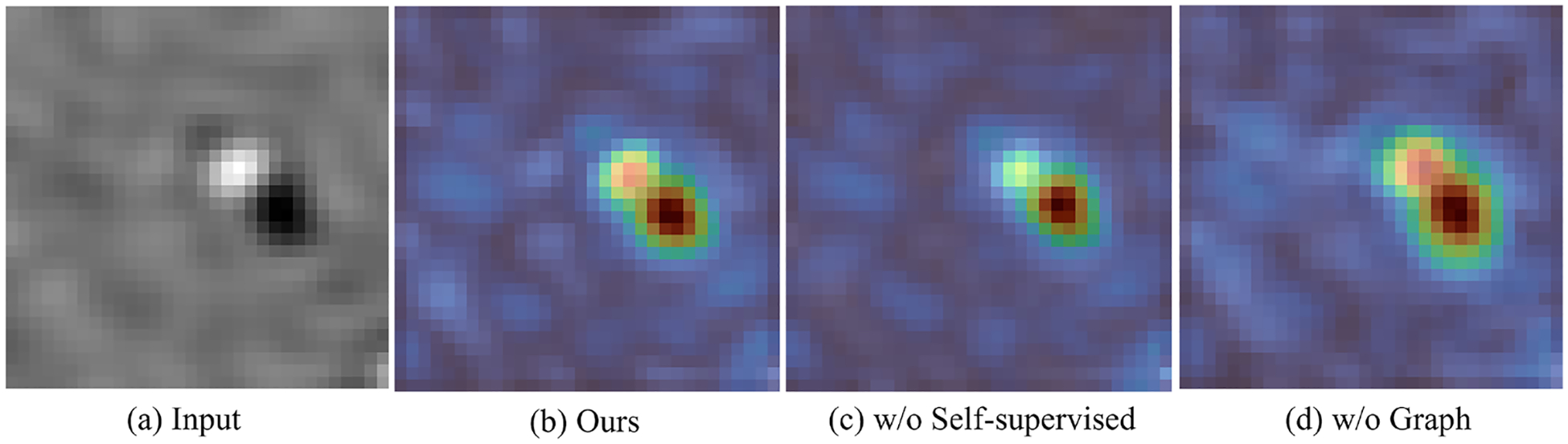
Heatmap visualization of feature activations in the ablation study of our graph design.

To validate the effectiveness of our proposed graph-guided attention mechanism and the contextual graph construction, we perform ablation study on the module design and report the results in Fig. 10. Specifically, we extract the feature map output from the encoder of our model, compute the voxel-wise activation strength by taking the channel-wise L2 norm, and normalize the resulting 3D heatmap to [0,1]. As shown in Fig. 10, removing the graph-guided attention (Fig. 10(d)) leads to weak and diffused activations, where the encoder fails to capture the particle boundaries. When the graph is used to guide attention (Fig. 10(c)), the encoder becomes more sensitive to boundary regions. Furthermore, by incorporating contextual graph construction to enhance the cross-view understanding, the boundaries become even more distinct and well localized (Fig. 10(b)).

##### Graph construction methods.

**Fig. 11.**
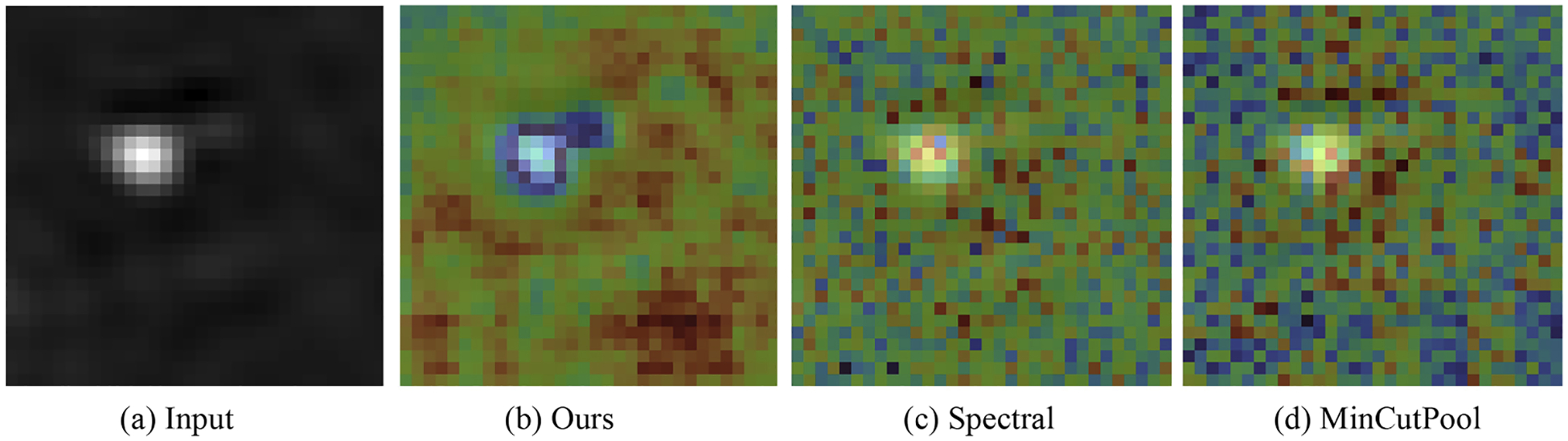
Heatmap visualization of feature activations in the ablation study comparing our k-means graph construction with spectral and mincutpool methods.

We set two different construction methods, spectral clustering and Mincutpool, as the ablation choice for ablation study. As shown in Table 9, K-means clustering achieves the highest mIoU (86.9%), while spectral and MinCutPool perform slightly worse, validating that our simple clustering strategy is already effective. We also provide qualitative visualizations of the learned feature activations under the same processing method as Fig. 10. For visualization, we overlay the normalized heatmap onto the corresponding grayscale input slices. As shown in Fig. 11(a) displays the original grayscale input slice; (b) shows our method, where the feature map activations (computed as the voxel-wise L2 norm across channels) are normalized and mapped back to the input space. Strong activations are clearly concentrated on the structural boundary of the target object. In contrast, the spectral clustering (c) and MinCutPool (d) baselines produce noisy and less localized activations.

##### Fusion scheme.

To validate the effectiveness of our fusion scheme choice, we choose two separate fusion methods to replace the direct summation used in our method as the comparison baselines. As can be seen from Table 9, direct summation of logits provides a strong baseline (86.9%), outperforming normalization or learnable weighting, which suggests that complex weighting is unnecessary.

##### Loss function design.

As can be seen from Table 9, using cross-entropy loss alone yields the best performance (86.9% mIoU). We set the Boundary Dice Loss as the boundary-aware loss, but its combination with CE does not bring further improvements (86.5%), while adding contrastive regularization even degrades the results (84.1%). This indicates that the proposed model, when trained with CE alone, already captures sufficient structural and boundary cues, and that additional loss terms may introduce redundant or noisy constraints that hinder optimization.

##### Decoder designs.

We validate the effectiveness of each decoder design in our method. We validate the effectiveness of each decoder design in our method. In Tables 2, 3, and 5, we additionally provide results using a simple MLP as the decoder (as “MVPGFormer (MLP)”), from which it can be observed that both of our proposed decoder designs achieve consistently superior performance. We replace the decoders of Swin UNETR, MedNeXt and SwiFT with our proposed decoder designs. As Swin UNETR and MedNeXt share a similar decoder design, for fair comparison, we also combine our transformer encoder with the decoder proposed in Swin UNERT as the “Base” framework and re-conduct experiments on the SHREC dataset. As show in Fig. 9(a), we present the mIoU scores for different combinations of methods and decoders. “Base” denotes the methods’ original framework without any changes, “MF” denotes the methods’ decoders are replaced with the MF and “P3DA” denotes the methods’ decoders are replaced with the P3DA. As our proposed MF is similar to their existing decoder design, which are all based on multi-layer feature fusion, the improvement brought about is not significant (refer to blue and red dashed lines in Fig. 9(a)). Our proposed P3DA significantly improves all methods’ performance, which demonstrates that the P3DA can effectively expand the receptive field and enhance the performance of the 3D transformer encoders (refer to green dashed lines in Fig. 9(a)).

##### Positional encoding design.

In addition to our default multi-view positional encoding, we conducted ablations with three common alternatives: learned 3D positional encodings, sinusoidal 3D encodings, and rotary encodings. As shown in Fig. 9(b), these variants yield only marginal differences compared to our design. This demonstrates that assigning distinct encodings to each orthogonal view already provides sufficient cross-view geometry, validating that our proposed encoding strategy is both effective and efficient without introducing extra parameterization.

##### Loss balancing.

Our training objective combines two terms: segmentation loss ${ℒ}_{\text{CE}}$ and reconstruction loss ${ℒ}_{\text{MSE}}$. Both are defined as voxel-wise averages, so their magnitudes are naturally comparable and no additional weighting coefficients are required. To verify robustness, we further conducted a sensitivity analysis by introducing two coefficients, $\lambda_{1}$ and $\lambda_{2}$, to balance the two losses. As shown in Fig. 9(c), the model achieves the best performance when $\lambda_{1}=\lambda_{2}=1.0$, while maintaining stable results across a wide range of values, confirming that our optimisation is not sensitive to the precise weighting scheme.

## 6. Conclusion

In this work, we introduce the first transformer-based framework Multi-View Perspective Transformer (termed as MVGFormer) for cryo-ET segmentation. Different from current CNN-based cryo-ET segmentation methods, we use a multi-view perspective fusion transformer encoder to enforce the model to focus more on the global information of the input data, thereby acquiring richer spatial information. Unlike other 3D vision transformer encoders, which only use the single view of the 3D image as the input and omit the multi-perspective observation information, our transformer encoder fuses feature embeddings from multi observation perspectives to fully leverage the characteristics of the 3D inputs. Additionally, two decoder designs are proposed to evaluate the effectiveness of our MVGFormer’s encoder feature representation. Extensive experimental results demonstrate that our MVGFormer set the new state-of-the-art on both cryo-ET tomogram and subtomogram datasets. For future work, we will curate more cryo-ET data, extend 2D prompting and clustering techniques, and propose a segmentation foundation model based on MVGFormer for cryo-ET to address biological challenges in real-world scenarios.

### Limitations of our work

The main issue with our approach is the increase in computational complexity without a significant improvement in effectiveness. This is because the transformer structure lacks strong inductive bias, thus requiring larger amounts of data support (for example, ViT [16] is pretrained on ImageNet22k). Therefore, we believe that the size of the dataset limits the performance of our method. In future work, we will focus on collecting more data to train the model. Meanwhile, we will also attempt to improve the model to enhance efficiency and reduce computational costs.

## Figures and Tables

**Table 1:** Summary of the proposed framework.

Dimension	Component	Function / Output
Input	3D input with XY, XZ and YZ projections	Orthogonal cryo-ET views providing complementary cues
Encoder	Transformer encoder with multi-view tokens	Captures cross-view semantic consistency
Fusion	Graph-based aggregation module	Models spatial and frequency-level relationships
Decoder	Multi-scale convolutional layers	Produces voxel-wise segmentation output
Learning Objective	View-masked SSL + CE loss	Jointly optimizes reconstruction and segmentation

**Table 2:** Experimental results on cryo-ET tomogram segmentation.

Method	mIoU	Dice
URFinder _[SHREC21]_	66.8	80.1
U-CLSTM _[SHREC21]_	77.6	87.4
MCDSNet _[SHREC21]_	78.4	87.9
YOPO _[SHREC21]_	68.1	81.0
CFN _[SHREC21]_	75.6	86.1
TM _[SHREC21]_	50.0	66.6
TM-F _[SHREC21]_	51.8	68.2
DeepFinder _[SHREC21]_	83.5	91.0
VoxResNet _[NeuroImage18]_	83.7_±0.4_	91.1_±0.3_
Swin UNETR _[CVPR22]_	79.4_±0.2_	88.5_±0.1_
MedNeXt _[miccai23]_	82.7_±0.6_	90.5_±0.3_
SwiFT _[NeurIPS23]_	81.5_±0.1_	89.8_±0.1_
MVGFormer(MLP)	84.6_±0.3_	91.6_±0.2_
MVGFormer(MF)	85.7_±0.1_	92.6_±0.2_
**MVGFormer(P3DA)**	**86.9**_±0.2_	**93.1**_±0.4_
MVGFormer(permutation)	86.8_±0.1_	93.0_±0.3_

We use the **bold** and the underlined to represent the best results and the runner-ups, respectively.

**Table 3:** Experimental results on cryo-ET subtomogram segmentation using simulated and real datasets.

Simulated dataset		
Method	mIoU	Dice
U-CLSTM _[SHREC21]_	76.8_±0.4_	87.2_±0.1_
DeepFinder _[SHREC21]_	81.0_±0.1_	89.5_±0.1_
VoxResNet _[Neurolmage18]_	84.3_±0.4_	91.4_±0.2_
Swin UNETR _[CVPR22]_	78.8_±0.9_	88.1_±0.6_
MedNeXt _[miccai23]_	82.7_±0.5_	90.8_±0.3_
SwiFT _[NeurIPS23]_	80.1_±0.1_	88.9_±0.7_
MVGFormer(MLP)	83.9_±0.4_	91.2_±0.2_
MVGFormer(MF)	84.9_±0.3_	91.9_±0.2_
**MVGFormer(P3DA)**	**87.1**_±0.2_	**93.2**_±0.2_
PolyGA dataset		
Method	mIoU	Dice
VoxResNet (w/o pre)	46.0_±1.5_	61.6_±0.9_
3D-UNet _[JBCB21]_	49.4_±0.2_	66.1_±0.1_
DeepFinder _[SHREC21]_	53.4_±0.3_	69.6_±0.4_
MedNeXt _[miccai23]_	55.2_±0.5_	71.1_±0.2_
VoxResNet _[Neurolmage18]_	59.8_±0.6_	74.8_±0.4_
SwiFT _[NeurIPS23]_	57.0_±0.2_	72.6_±0.3_
MVGFormer(MLP)	61.5_±0.1_	76.2_±0.3_
MVGFormer(MF)	62.3_±0.1_	76.8_±0.4_
**MVGFormer(P3DA)**	**63.7**_±0.2_	**78.5**_±0.3_
Erwinia dataset		
Method	mIoU	Dice
VoxResNet (w/o pre)	72.4_±0.7_	80.3_±1.1_
3D-UNet _[JBCB21]_	76.7_±0.2_	86.8_±0.3_
DeepFinder _[SHREC21]_	88.0_±0.3_	93.6_±0.2_
MedNeXt _[miccai23]_	89.4_±1.1_	94.4_±0.1_
VoxResNet _[Neurolmage18]_	95.4_±0.1_	97.6_±0.1_
SwiFT _[NeurIPS23]_	91.7_±0.3_	95.7_±0.1_
MVGFormer(MF)	96.8_±0.2_	98.4_±0.2_
**MVGFormer(P3DA)**	**97.9**_±0.4_	**98.9**_±0.1_

We use the **bold** and the underlined to represent the best results and the runner-ups, respectively.

**Table 4:** Experimental results on cryo-ET tomogram segmentation (SHREC dataset) after pre-training on a simulated subtomogram dataset.

Method	mIoU	Dice
DeepFinder	83.6	91.3
MedNeXt	83.1	90.8
VoxResNet	85.3	91.4
SwiFT	82.8	91.0
MVGFormer(MF)	89.0	94.2
**MVGFormer(P3DA)**	**91.1**	**95.1**

**Table 5:** Experimental results for particle picking using EMPIAR-10499 and CZII dataset.

	EMPIAR-10499			CZII		
Method	Precision	Recall	F_1_	Precision	Recall	F_1_
DeepFinder _[Nat. Methods21]_	50.5	51.7	52.7	72.1	71.7	71.9
EMAN2 _[J. Struct. Biol07]_	26.1	55.3	35.5	54.7	64.7	59.3
crYOLO _[Commun. Biol.19]_	47.8	56.8	52.0	63.8	65.6	64.7
VoxResNet _[NeuroImage18]_	57.1	71.4	64.7	74.0	73.0	73.5
SwiFT _[NeurIPS23]_	54.0	69.4	63.5	67.6	58.1	62.5
MVGFormer(MLP)	58.7	57.7	58.2	75.6	74.4	75.0
MVGFormer(MF)	59.4	75.0	66.8	76.0	77.8	76.9
**MVGFormer(P3DA)**	**61.2**	**78.4**	**69.8**	**78.3**	**81.6**	**79.9**

We use the **bold** and the underlined to represent the best results and the runner-ups, respectively.

**Table 6:** Zero-shot learning performance for particle picking using EMPIAR-10499 and CZII dataset.

	EMPIAR-10499			CZII		
Method	Precision	Recall	F_1_	Precision	Recall	F_1_
VoxResNet	48.5	46.3	47.4	64.3	56.6	60.2
SwiFT	43.8	62.8	51.6	54.7	64.7	59.3
**MVGFormer(MF-ZS)**	**53.2**	**63.5**	**57.9**	**69.6**	**63.7**	**66.5**
MVGFormer(P3DA-ZS)	50.3	57.6	53.7	67.1	60.8	63.8
MVGFormer(MF-Fully)	59.4	75.0	66.8	76.0	77.8	76.9
MVGFormer(P3DA-Fully)	61.2	78.4	69.8	78.3	81.6	79.9

We use the **bold** and the underlined to represent the best results and the runner-ups, respectively.

**Table 7:** Inference time and computational complexity comparison.

Method	Param.	Speed	secs/Epoch	Training hours
VoxResNet	6.8M	31fps	182	5.06h
MedNeXt	17.6M	9fps	1320	36.67h
SwiFT	10.7M	15fps	269	7.47h
**MVGFormer(P3DA)**	13.7M	25fps	908	25.22h

**Table 8:** Ablation study on the hyper-parameters of our proposed model on SHREC dataset.

C	mIoU	Dice	$L_{n}$	mIoU	Dice	$H_{p}\times W_{p}\times D_{p}$	mIoU	Dice
128	80.5	89.2	6	76.8	86.9	2×2×2	82.3	90.2
**256**	**86.9**	**93.1**	**12**	**86.9**	**93.1**	**4**×**4**×**4**	**86.9**	**93.1**
512	82.3	90.2	18	78.7	88.1	8×8×8	71.5	83.3
1024	84.1	91.3	24	81.6	89.9	16×16×16	54.8	70.8

$C,L_{n}$ and $H_{p}\times W_{p}\times D_{p}$ denote the size of the hidden dimension, the number of the transformer layer and the patch size, respectively.

**Table 9:** Ablation study on the cluster number K, graph construction methods (G.C.M.), fusion schemes and loss function of our proposed model on SHREC dataset.

K	mIoU	G.C.M.	mIoU	Fusion Schemes	mIoU	Loss Function	mIoU
8	83.6	spectral	85.3	norm + sum	85.7	${ℒ}_{\text{CE}}+{ℒ}_{\text{boundary}}$	86.5
16	**86.9**	k-means	**86.9**	sum	**86.9**	${ℒ}_{\text{CE}}$	**86.9**
32	84.7	MinCutPool	83.9	learnable-weight	86.4	${ℒ}_{\text{CE}}+{ℒ}_{\text{CL}}$	84.1

**Table 10:** Ablation study on the cluster number K of our proposed model on SHREC2021, PolyGA and EMPIAR-10499 dataset.

	SHREC2021		PolyGA		EMPIAR-10499		
K	mIoU	Dice	mIoU	Dice	Precision	Recall	F_1_
8	83.6	91.2	59.1	74.3	58.7	75.2	65.9
12	84.7	91.7	60.2	75.9	59.1	76.0	66.5
**16**	**86.9**	**93.1**	**63.7**	**78.5**	**61.2**	**78.4**	**69.8**
20	86.5	92.7	62.0	76.5	60.3	77.3	67.8
24	85.3	92.0	61.4	76.1	59.5	76.1	66.8
28	84.6	91.6	61.5	76.3	59.7	76.2	67.0
32	84.7	91.7	60.9	75.7	58.6	74.6	65.7

**Table 11:** Ablation study of the proposed multi-view perspective fusion strategy (MVP) in our framework.

Method	mIoU	Dice
Swin UNETR + MVP	81.6 ↑ (79.4)	89.8 ↑ (88.5)
MedNeXt + MVP	83.9 ↑ (82.7)	91.2 ↑ (90.5)
SwiFT + MVP	83.4 ↑ (81.5)	91.0 ↑ (89.8)

We apply the proposed strategy on three recent 3D transformer-based approaches and re-conduct the experiments on SHREC dataset. The values inside the parentheses are the original results w/o MVP.

**Table 12:** Ablation study of the proposed multi-view perspective fusion strategy (MVP) using each perspective as an independent primary viewpoint.

Method	mIoU	Dice
MVGFormer(XY)	82.2	90.2
MVGFormer(YZ)	82.7	90.8
MVGFormer(XZ)	81.9	90.0
**MVGFormer(P3DA)**	**86.9**	**93.1**

**Table 13:** Ablation study of the proposed multi-view perspective fusion strategy (MVP) using repeated single-view inputs with distinct positional embeddings.

Method	mIoU	Dice
MVGFormer(XY*3)	82.4	90.3
MVGFormer(YZ*3)	83.1	90.5
MVGFormer(XZ*3)	82.6	89.9
**MVGFormer(P3DA)**	**86.9**	**93.1**

**Table 14:** Feature diversity across different views.

view-pair	Cosine Similarity ↓	Feature Diversity ↑
XY & YZ	0.61	0.81
XY & XZ	0.67	0.75
XZ & YZ	0.74	0.73

## Data Availability

Data will be made available on request.

## Appendix A. Dataset description

In this section, we provide more details about the dataset we used to conduct the experiments.

### A.1. Tomogram dataset

As aforementioned, each tomogram contains particles from 13 proteins plus vesicle and fiducial. Here we list all the categories of the proteins: **4V94, 4CR2, 1QVR, 1BXN, 3CF3, 1U6G, 3D2F, 2CG9, 3H84, 3DL1, 3QM1, 1S3X** and **5MRC**. Other than particles, the background consists mostly of water and structural noise.

### A.2. Subtomogram dataset

Cryo-ET subtomogram segmentation is a binary segmentation task. The simulation process we used, however, can only generate grey-scale ground-truth masks. Hence, following [24], we use a threshold to turn the grey-scale mask into a binary one. The threshold we used in our paper is set to 300. As mentioned in Section 5.1.1, the simulated subtomogram dataset is simulated from 50 macromolecules. We provide all the macromolecules used for simulation in Table A.15.

**Table A. 15:** Macromolecules contained in simulated cryo-ET subtomogram dataset. Each macromolecule is simulated with three different noise levels.

Macromolecules									
1bxn	1c5u	1cf5	1e0p	1f1b	1hak	1hwn	1hwo	1o6c	1qoq
1vim	1viy	1yg6	1z30	2ane	2byu	2fuv	2h12	21db	2oqa
2pcr	2qes	2wiz	2z04	2zzw	3g11	3hhb	3ku0	3le7	4d4r
4dxy	4fb9	4leh	4yov	5ddz	5wde	5wvm	5x1i	5xls	6afk
6i7c	6jaw	6jhh	6jhi	6jvz	6lov	6loy	6oo1	6t3e	7cgt
